# Asylums of the World. IV.—The Construction of Asylums

**Published:** 1892-07-30

**Authors:** 


### The Institutional Workshop.

### INSTITUTION LITERATURE.

### ASYLUMS OF THE WORLD.

PART IV.—THE CONSTRUCTION OF ASYLUMS.

Volume II. of the work before us is chiefly concerned with this part of the subject, and, so far as we can judge, nothing is left out which it was desirable to deal with.

THE CLASSIFICATION OF PATIENTS.—We hold that no asylum is properly built which does not fully provide for an absolute classification or division between four classes of patients, namely, the recent acute cases, the chronic industrious insane, the noisy, dirty, or destructive chronic insane, and the sick and infirm. While the structural arrangements for the separation of these different classes of patients should be as rigid as possible, it will still rest with the management to dilute the different classifications with patients from other divisions should they see fit to do so, but no effort should be spared by the builders to give facility for absolute classification. The reasons for this are so obvious, and the principle has been adopted in so many modern asylums, that it is unnecessary to discuss them.

We are particularly pleased with the deliverance upon the vexed question of asylum farms. The opinion is one that the writer of this review has held for many years, and it is so strongly and succinctly stated here that we reproduce it: "For an asylum of medium size, say about 600 beds, 50 or 60 acres will be found amply sufficient, while 100 acres will be enough for 1,000 beds or upwards. A large estate is simply a source of trouble and expense to all concerned, and it rarely or never means that a larger number of patients is employed upon the farm; rather, indeed, it means the opposite, for it induces the committee to embark on extensive farming operations by extra paid hands, whereas an increased number of patients should mean an increase of spade cultivation, and for this purpose a very few acres are sufficient."

We consider this statement to be wholly unanswerable. Our own experience has been that where an asylum possesses a large farm there is always a tendency towards that condition of separate interests in which the farm benefits more from its connection with the asylum than the patients do from the farm. The erroneous opinion held by the members of some asylum committees, who imagine that large farms are necessary for the employment of lunatics, is worth investigation by those gentlemen themselves, and deserving of exposure in the interests of asylums.

The importance of building an asylum near a good and unfailing water supply, the importance of providing for future extension, and the necessity of securing a site which permits of free and easy drainage, are insisted upon. With regard to the latter point, the new international sewage purification system, in use in one or two asylums in the Stirling District Asylum, Scotland, and in the East Riding Asylum, Beverley, Yorks, is worthy of notice, as it can be adopted upon any site and in any kind of soil. It is a further advantage, as is pointed out in the work in question, to build an asylum near a large town, for thereby not only are business arrangements facilitated, but the comfort and convenience of the staff is increased.

With regard to the possibility of rendering a large institution for the insane homelike, much might be said from opposite points of view. We have no desire to indulge in argumentative disquisitions in reply to those who affirm that an asylum is incapable of being by a process of upholstering converted into anything resembling home life, or that patients can by this means be deluded into considering themselves differently situated from what they really are. We would only refer to some asylums where much has been done in the direction of breaking down the institution idea by means of comfortable furnishing, and by floral and permanent decoration; and on the other hand refer them to asylums, if there are such, where nothing has been done in this respect, and ask them to judge between the two.

If an institution for the insane is bare and cheerless and badly furnished, the prison idea is forced into prominence. The more the walls are decorated, the more green plants are scattered throughout the wards and corridors, and the more the floor spaces are judiciously broken up with tables, chairs, pianos, or benches, by so much the more will the prison gloom be supplanted by a cheerful savour of comfort, and by the ideas that cluster round the human conception of the home and the beautiful.

The internal fittings and arrangements of an asylum are often of more importance in many respects than its outward configuration.

As little wood as possible, and that of the hardest and most durable kind, should be used in the interior, and its use should, we think, be limited to the necessary fittings, such as those of the doors and windows, and to the flooring of the day-rooms and sleeping apartments.

The corridors should be tiled both floor and dado, the wards, if not provided with an ornamental tile dado, should possess one of the hardest wood such as oak, and the sleeping apartments may have a dado of Keane's cement.

We are strongly of opinion that no wood should form any part of the internal fittings of the lavatories or w.c.'s (except in the closet seats), and that these apartments should be plainly but substantially tiled from floor to ceiling. With fairly good sanitary arrangements, in conjunction with the principles regarding the internal construction which we have laid down in the immediately preceding sentences, an asylum should be as near sanitary perfection as modern knowledge can make it. We have laid great stress upon the material employed in the internal fittings, because it has been our misfortune to have to do with asylums in which it is not too much to say that the emanations from the bodies of successive generations of lunatics seemed to have permeated the structure through to the stone and lime. An annual expensive coat of paint was employed to assist in the further destruction of the wood-work.

In Volume I., if we mistake not, the subject of general dining-rooms for patients is not enthusiastically advocated. This seems a pity, for nothing appears more necessary than that the patients should enjoy the variety of going to and from the dining-room, while their wards are aired in their absence.

Imagine a succession of wet days, during which these unfortunate people remain in their wards staring at the same objects and tormented by troublesome neighbours without a break for twelve long hours daily.

A strict adherence to those hygienic and sanitary laws, which are more especially applicable to institutions where large numbers of persons are aggregated, should influence the external form of asylum buildings, in so far as they require that the buildings shall be so disposed as to admit of a sufficiency of fresh air and sunlight penetrating into every corner and nook.

For this end the styles of asylums called in the work before us pavilion and corridor asylums are the best. Excellent plans of several of the more modern asylums are given in this book. Chief of these are Claybury in Essex, the new Gloucester County Asylum, and the City of Glasgow District Asylum at Gartlock in Lanarkshire. The last named, like the other new Scottish asylums and some of the old ones, possesses the admirable adjunct of a separate hospital block for the treatment of acute and recent cases.

Of the supreme importance of such an arrangement too much good cannot be predicated. The system is entirely due to the enlightened views of the Scottish Lunacy Commission.

The work just reviewed is bulky and contains an immense amount of reading, and after a conscientious survey we feel justified in stating that there is nothing contained within its boards which is not only worth reading, but to all who are connected with asylums worth studying and remembering. As a book of reference for medical officers of asylums, asylum directors, and asylum architects, as well as for those interested in lunacy legislation, these two volumes will be found invaluable. They should occupy a prominent place in the committee room of every British asylum. So far as we know it is the first real attempt to place before the English-speaking public a reliable store book of information upon asylums and all lunacy matters in every civilised country in the world.

If the two volumes upon hospitals, which we believe are shortly to appear to complete this great and laborious undertaking, subserve as useful a purpose as the two volumes upon asylums promise to do, Mr. Burdett will have earned the deserved thanks of all scientific, medical, and philanthropic workers.

